# The Effect of Feedback and Operational Experience on Children’s Rule Learning

**DOI:** 10.3389/fpsyg.2017.00534

**Published:** 2017-04-13

**Authors:** Fuhong Li, Liufang Xie, Xue Yang, Bihua Cao

**Affiliations:** ^1^School of Psychology, Jiangxi Normal UniversityNanchang, China; ^2^School of Psychology, Southwest UniversityChongqing, China

**Keywords:** balance scale task, rule levels, learning, feedback, children

## Abstract

This study aimed to examine the relative effect of feedback and operational experience on children’s rule learning in a balance scale task, in which 88 children under the age of 7 years were asked to judge the state of equilibrium under four conditions. In the *Control* condition, children were required to observe the scale and predict which side would tilt down or keep balance, without feedback on the correctness of their answer. In the *Operation* (*Op*) condition, children were required to place the weights on the scale just like the experimenter did before they made predictions. In the *Feedback* (*Fe*) condition, feedback was provided for each prediction, but children were not allowed to operate the scale. In the *Op-Fe* condition, children could operate the scale and they were provided feedback for each prediction. The results showed that, (1) children in *Control* condition merely adopted the lowest level of rule, the Weight Rule; (2) when they were either given feedback or the opportunity to operate the scale, they used a higher level rule, such as the Distance Rule, more frequently; and feedback was more effective than the operational experience was in promoting rule learning; (3) when they were allowed to operate the scale, and were simultaneously provided feedback, rule learning increased markedly, suggesting that feedback-based operation is the most efficient method for facilitating children’s rule learning.

## Introduction

The balance scale task is one of the classical paradigms that has been used to examine the validity of various theories on cognitive development (Siegler, 1976; Karmiloff-Smith, 1995; Shultz and Takane, 2007; Bonawitz et al., 2012; Hofman et al., 2015). During the task, objects with different weights were put onto two sides of a scale, and children were required to judge which side would tip down (Siegler et al., 1981; Siegler, 2005; Schapiro and McClelland, 2009). It has been demonstrated that children of different ages will use different rules that are classified into levels to complete the balance scale task (Halford et al., 2002; Jansen and van der Maas, 2002; Siegler, 2005; Schrauf et al., 2011).

Overall, the rules used by children could be classified into the following four levels that are ordered hierarchically (Siegler, 1976, 2005; Siegler et al., 1981). Rule I, the Weight Rule, is based on weights that are placed on both sides of a balance scale, irrespective of distance. With Rule II (Distance Rule), the Weight Rule is correctly used, and distance is considered if the weights are first found to be equal. With Rule III, children consider two dimensions, which are the weights and distance from the fulcrum, when there is a conflict between these two dimensions. For example, when one side has more weight but less distance than the other side, children will not understand how to judge the situation. With Rule IV, children consider the two dimensions, and use the equation: weight 1 × torque 1 = weight 2 × torque 2. Previous studies have demonstrated that children under the age of 8 years initially adopt the Weight Rule (Siegler et al., 1981; Siegler, 2005). These studies suggested that, the older the children are, the more likely they are to use rules of higher levels. Jansen and van der Maas (1997) conducted a latent class analysis of balance scale performance with 484 children. They confirmed the existence of Siegler’s Rules I, II, and IV, and suggested that alternative rules, based on the addition of weight and distance or “compensation rules” (Normandeau et al., 1989; van Maanen et al., 1989), should be incorporated into the rule system.

A number of studies attempted to address how to improve children’s understanding and use of the higher level rules, such as the Distance Rule and Rule III (Siegler, 1976; Siegler and Chen, 1998, 2002; Jansen and van der Maas, 2001; Halford et al., 2002; Pine et al., 2004; Schrauf et al., 2011; Legare and Lombrozo, 2014). Some investigators showed that presenting children with distance items with extreme distance difference between the two sides of scale leads to more usage of Rule II (Jansen and van der Maas, 2001). Other investigators have demonstrated that rewards or feedback can help children learn the higher rules (Siegler and Svetina, 2002; Hofman et al., 2015). For example, Hofman et al. (2015) found that, in a Math Garden game, presenting feedback and rewards leads to more Rule II behavior. Siegler and his colleagues demonstrated that, when 5- and 8-year-olds who initially used Rule I were presented with feedback on problems which included solutions that demanded the application of Rule II or Rule III, the 8-year-olds were more likely to form such rules (Siegler, 1976). In Siegler and Chen‒ (1998) study, 4- and 5-year-olds were presented with distance problems, and a pretest–feedback–posttest design was used. In each of the 16 trials during the feedback phase, each child was required to predict which side of the scale (if either) would tip down if the supporting lever were released. Following the child’s judgment, the supporting lever was released and the child observed the scale’s movement. Finally, the child was required to provide an explanation. Their results indicated that the children’s performance on the distance problem was influenced by feedback-based learning. Children’s performance increased gradually within the trials of the distance problem. However, it should be noted that children were repeatedly presented with the same type of problems in a block; that is, children received intensive training to solve the same type of problems, such as distance problems, in a block.

The purpose of the present study was to explore whether feedback can effectively improve children’s spontaneous rule learning when the various types of problems were equally presented in a block. Children are able to acquire a rule through inductive reasoning (Li et al., 2009, 2017; Schulz, 2012) or reversal learning (Harlow, 1949). In the balance task, when children were repeatedly presented with the same type of distance problems, they acquired the higher level rule based on the feedback (Siegler, 1976; Siegler and Chen, 1998). However, if children are not presented with the same type of problem, they would not experience intensive training. Therefore, it would be interesting to examine if they would still receive some benefit from the feedback, as seen in the previous studies (Siegler, 1976; Siegler and Chen, 1998). Thus, we presented children with five types of problems randomly, and each type of problem appeared equally throughout the assessment. After they provided a prediction for each problem, we provided them with feedback. The distance problems were mixed with other types of problems; we tentatively predicted that feedback might also be helpful for their understanding of the higher level rules, because of the fundamental effect of feedback-based learning in children’s cognitive development (Sloutsky and Fisher, 2004; Peters et al., 2014), and the computational models of the balance scale task predict that feedback will lead to switches from Rule I to Rule II (van Rijn et al., 2003; Schapiro and McClelland, 2009; Hofman et al., 2015).

Another potential approach to enhance children’s rule learning is to allow them to operate the balance scale themselves (Siegler, 1976; Halford et al., 2002). In the study by Siegler (1976), for example, children in the experimentation group were told that there were rules by which they could know which way the balance would tip, and that they should “experiment” by placing the weights on the pegs, in as many different ways as they needed, to learn how balancing worked. Siegler (1976) found that the acquisition of the higher level rules, such as the Distance Rule, was not improved when children were allowed to operate the balance scale themselves, or when they were guided by the experimenter. Siegler (1976) indicated that a major unanticipated finding in Experiment 1 was that the experimentation treatments did not produce a greater movement toward Rule IV. In fact, children did not show increasing usage of Rule II in Experiment 1. Only after intensive training in the distance problem (12 trials in Experiment 2) did the 5-year-old children show significant enhancement in their use of Rule II, the Distance Rule.

Some studies suggested that manual operation can improve children’s rule learning (Piaget, 1964; Trawick-Smith, 2013; Crain, 2015), and we presumed that manual operation can promote children’s acquisition of Rule II; however, it is still unclear whether the manual operation is more or less effective than feedback is for children to learn the Distance Rule. The answer to this question may help us comprehend the underlying mechanism of the transition from Rule I to Rule II. Because both manual operation and feedback-based observational learning are the primary approaches of learning for children, they function differently in promoting children’s learning of the Distance Rule. The former helps children to encode the distance information more precisely (Siegler and Chen, 1998), while the latter can help children understand the role of the distance factor in balancing the scale. Some researchers suggested that, for children, the failure of transition from Rule I to Rule II is due to the fact that they may have difficulty in processing distance information (Siegler, 1976; Siegler and Chen, 1998). If it is true, allowing children to operate the scale and helping them to precisely encode distance information can enhance children’s performance in solving the distance problem. Alternatively, we assumed that the understanding of the role of distance in balancing is rather important for children. That is, knowing the function of a factor is more helpful than knowing the precise encoding of that factor. Therefore, we hypothesized that feedback might be more effective in promoting children’s rule learning than operational experience would be.

## Materials and Methods

### Participants

Ninety children (46 boys and 44 girls) participated in the experiment. They were divided into four groups to complete the tasks. Their average age was 5 years and 2 months, ranging from 4 years and 3 months to 6 years and 1 month (*SD* = 11 months). All children were mentally healthy, and they had normal eyesight and hearing. All subjects’ parents or teachers provided both verbal and written consent for participation of this study, and the conduction of the study was approved by the IRB of Jiangxi Normal University (China). Ethical approval for this study was obtained from the ethics committee of Liaoning Normal University (China). Three children failed to finish all the problems, so the data of those children were excluded from the analyses.

### Materials and Design

The materials were two wooden balance scales, 20 ring-shaped metal disks, and four paper blocks. Each balance scale’s arm was 20 inch long, with four pegs on each side of the fulcrum. Each peg was of the same weight, height, and thickness. The first peg on each side was 2.5 inch from the fulcrum, and each subsequent peg was 2.5 inch from the preceding one. The arm of the scale could either tip left or right, or remain level, depending on how the metal disks were placed. Each disk weighed about 0.8 ounces, measured 0.5 inch in diameter, and had a hole in its center to fit on the pegs. The scale was held steady by two paper blocks, with one placed beneath each end. In order to avoid the mutual influence caused by the differences between experimental conditions, we randomly arranged the 88 children into the four experimental conditions described below.

#### No Feedback and No Operation (Control)

In this condition, children were neither provided with feedback nor with the opportunity to operate the scale. The experimenter presented each problem to the children and asked them to predict which side would tip down or remain level if the supporting blocks were removed. Children were not allowed to operate the scale. Additionally, they were not provided any feedback after they provided their answers.

#### Operation (Op)

Children were allowed to operate the scale, but no feedback was provided. The procedure was similar to that used in the *Control* condition, with the following exception. First, the experimenter would present another scale in front of the children. After the experimenter displayed a balance scale problem, the children were required to place the weights on the scale just like the experimenter did. After children finished the replication, they were required to predict what would happen to the scale when the supporting blocks on both sides were removed. The experimenter recorded the answer provided by the children, then began the next problem, without providing any feedback for each problem.

#### Feedback (Fe)

Feedback was provided for each problem, but children were not allowed to operate the scale. The procedure was similar to that used in the *Control* condition, except that the children were allowed to observe the movement of the scale after the two supporting blocks were removed. Simultaneously, the experimenter would say, “Now let’s see the correctness of your prediction.” If a prediction was correct, the experimenter would say, “Good, you got one point!” Otherwise the experimenter would say, “Oho, no! Let’s try the next trial.”

#### Operation and Feedback (Op-Fe)

Children were provided with both the feedback and the opportunity to operate the scale. The procedure was similar to that used in the *Op* condition, with the following exception. The experimenter would ask the children to remove the supporting blocks themselves to observe what would happen.

**FIGURE 1:**
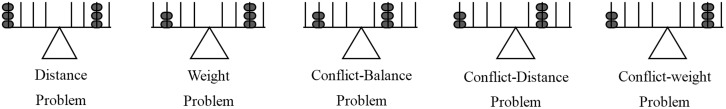
**Different types of balance scale problems**.

As shown in **Figure 1**, the balance scale problems consisted of five types: the distance problem, weight problem, balance problem, conflict-distance problem, and conflict-weight problem. There were five problems for each type, with 25 problems in total. The balance problems could further be divided into two sub-types: one in which both the weight and distance from the fulcrum were completely the same (i.e., the weights used on the balance scale were the same, and the distance from the fulcrum was the same) and the other in which they were all different (i.e., the weights used on the balance scale were different, and the distances from the fulcrum were different), but the scale remained level. Given that the former sub-type is too simple, we only used the latter.

### Procedure

Children were tested individually in a quiet room in a normal kindergarten, and her or his classmates were not allowed to see her or him during testing. First, an experimenter introduced the balance scale and explained the tasks explicitly to the children. Then the children were required to answer each problem and the experimenter recorded the children’s answers. The children would receive one point if their answer was correct (otherwise zero points). Different types of problems were presented to children randomly (Siegler, 1976; Halford et al., 2002). Owing to the random order of presentation, some children could face the most difficult problem, such as the conflict problems, at the beginning and fail. In the *Control* and *Op* condition, no feedback was provided for each response, so that the child would not experience negative emotions. When a child was unsure about the answer, he/she was encouraged to make a guess. In the *Fe* and *Op-Fe* conditions, a child could get frustrated on receiving negative feedback, and he/she may not want to do the next test. On such occasions, the experimenter would comfort and encourage him/her to get better during the next trials. The mean time needed to complete the task was about 20–30 min, and it was longer for young children in the *Op-Fe* condition.

## Results

A preliminary analysis indicated that there was no effect of sex on the prediction of all five types of problems in each condition. So the sex variable was not included in the following analyses. First, we conducted a repeated-measures ANOVA on the mean number of correct predictions by children to reveal the comparable effect of condition (Siegler, 1976; Siegler and Chen, 1998; Pine et al., 2004; Schrauf et al., 2011). The mean number of correct responses has been shown in **Table 1**. A 4 (condition) × 5 (problem type) ANOVA showed that the condition effect was significant, *F*(3,84) = 29.9, *p* < 0.001, η^2^ = 0.52. Multiple comparisons using the Bonferroni correction (the same was used for all multiple comparisons reported below) showed that the mean number of correct predictions in the *Op-Fe* condition was significantly larger than that in the other three conditions was (all *p*s < 0.001). More correct responses were found in the *Fe* condition than in the *Op* condition, and the difference was significant at the 0.05 level (*p* = 0.029).

**Table 1:** **The mean number of correct predictions by children at different condition**.

	Distance	Weight	Conflict-balance	Conflict-distance	Conflict-weight
*Control* (*n* = 25)	0.72 (1.31)	4.48 (1.16)	1.00 (0.29)	0.70 (1.49)	4.23 (1.52)
*Op* (*n* = 22)	1.45 (1.14)	3.59 (1.29)	0.82 (1.37)	0.91 (1.15)	3.36 (1.33)
*Fe* (*n* = 20)	2.45 (1.57)	4.08 (0.71)	1.15 (0.81)	0.81 (1.02)	3.50 (1.00)
*Op-Fe* (*n* = 20)	4.35 (1.22)	4.75 (0.72)	1.30 (0.80)	2.40 (0.88)	3.05 (1.09)

*The data in bracket is SD*.

The main effect of problem types was significant, *F*(4,332) = 118.2, *p* < 0.001, η^2^*=* 0.59. Multiple comparisons showed that the number of correct responses to the weight problem was significantly larger than it was for the other four types of problems (all *p*s < 0.001); the correct responses to the conflict-weight problem were significantly more frequent than were those to the distance problem, balance problem, and conflict-distance problem (all *p*s < 0.001). The correct responses for distance problem were significantly more frequent than were those for the balance problem and conflict-distance problem (all *p*s < 0.001). The correct responses for the balance problem did not significantly differ from those for the conflict-distance problem (*p* = 1.0).

**FIGURE 2:**
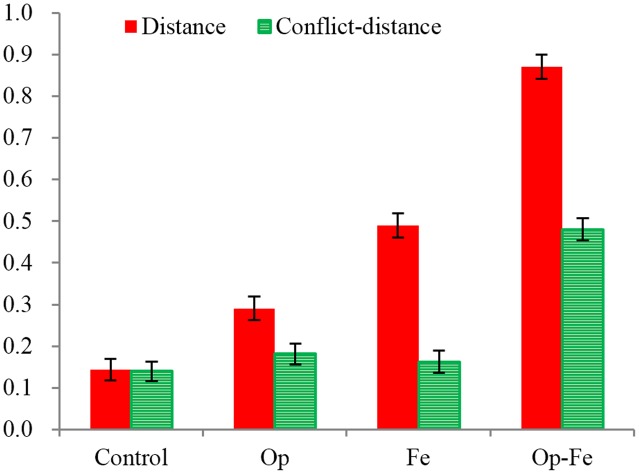
**Effects of the condition on the *distance* and *conflict-distance* problems**.

The interaction between the condition and problem type was significant, *F*(12,332) = 9.3, *p* < 0.001, η^2^ = 0.25. A simple effect analysis revealed the condition effect for all types of problems [*F_distance_*(3,83) = 30.81, *p* < 0.001, η^2^ = 0.53; *F_weight_*(3,83) = 5.22, *p* < 0.01, η^2^ = 0.16; *F_conflict-distance_*(3,83) = 9.53, *p* < 0.001, η^2^ = 0.26; *F_conflict-weight_*(3,83) = 3.59, *p* < 0.05, η^2^ = 0.12], with an exception for the balance problems [*F_balance_*(3,83) = 1.13, *p* = 0.34, η^2^ = 0.04]. For the distance problem (**Figure 2**), multiple comparisons revealed more correct responses in the *Op-Fe* condition than in the other three conditions (all *p*s < 0.001); the correct responses in the *Fe* condition were more frequent than they were in the *Control* condition (*p* < 0.001); and the number of correct responses in the *Op* condition did not significantly differ from those in the *Control* and *Fe* conditions (both *p*s > 0.05). For the weight problem, multiple comparisons revealed less correct responses in the *Op* condition than those in the *Control* condition and *Op-Fe* conditions (both *p*s < 0.05). For the conflict-distance problem (**Figure 2**), multiple comparisons revealed more correct responses in the *Op-Fe* condition than those in the other three conditions (all *p*s < 0.01). For the conflict-weight problem, the correct responses in the *Op-Fe* condition were significantly less frequent than they were in the *Control* condition (*p* < 0.02). Conditional effects were not found for the balance problem.

A simple effect analysis revealed the effect of problem type in all conditions [*F_Control_*(4,96) = 52.71, *p* < 0.001, η^2^ = 0.69; *F_Op_*(4,84) = 20.94, *p* < 0.001, η^2^ = 0.50; *F_Fe_*(4,76) = 39.70, *p* < 0.001, η^2^ = 0.68; *F_Op-Fe_*(4,76) = 47.65, *p* < 0.001, η^2^ = 0.72]. In the *Control* condition, a multiple comparison showed that the number of correct responses in the weight and conflict-weight problems did not significantly differ from each other (*p* = 1.0), but the correct responses to these two types of problems were significantly more frequent than they were to the other three types of problems (all *p*s < 0.001), and no significant difference was found between the other three types of problems (all *p*s > 0.05). In the *Op* condition, multiple comparisons revealed the same pattern of results as that observed in the *Control* condition. In the *Fe* condition, multiple comparison results showed that children made significantly more correct responses for the weight problem than those for the distance, balance, and conflict-distance problems (all *p*s < 0.01). The correct responses to the conflict-weight problem were significantly more frequent than they were to the balance and conflict-distance problems (all *p*s < 0.001). The correct responses to the distance problem were significantly more frequent than they were to the balance and conflict-distance problems (all *p*s < 0.05). In the *Op-Fe* condition, multiple comparison showed that there was no significant difference between the distance problem and weight problem (*p* = 0.88), but the correct responses to these two problems were significantly more frequent than were those to the other three types of problems (all *p*s < 0.05). The correct responses to the conflict-weight problem were not significantly different from those to the conflict-distance problem (*p* = 0.28), but the correct responses to these two problems were significantly more frequent than they were to the balance problem (all *p*s < 0.01).

**FIGURE 3:**
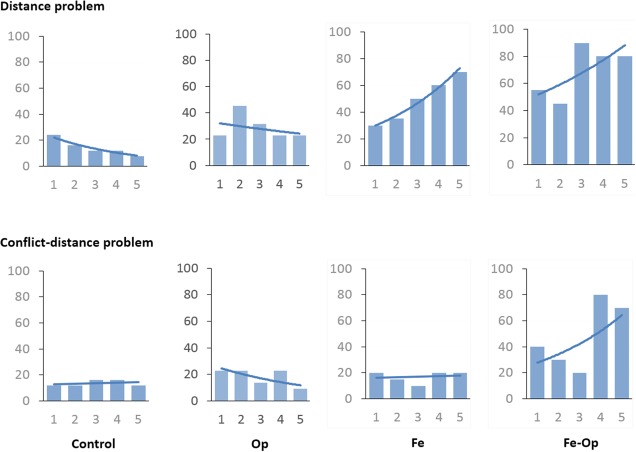
**Performance curves for the *distance* and *conflict-distance* problems under all conditions.** The numbers on the horizontal axis denote the order of the tests, and the numbers on the vertical axis denote the proportion of correct responses.

The above analysis showed that the condition effect was mainly reflected in the distance and conflict-distance problems. In order to further investigate the potential effect of learning on these problems, we analyzed the changing accuracy across trials for each type of problem, by performing a logistic regression analysis. As shown in **Figure 3**, the results showed that there was no learning effect in the *Control* and *Op* conditions; that is, children did not increase the accuracy with the number of trials. However, in the *Fe* condition, there was a learning effect for the distance problem (β = 0.448, *p* < 0.01). There was no learning effect for the conflict-distance problem. In the *Op-Fe* condition, the learning effect appeared for both distance (β = 0.661, *p* = 0.011) and conflict-distance problems (β = 0.467, *p* < 0.01).

**FIGURE 4:**
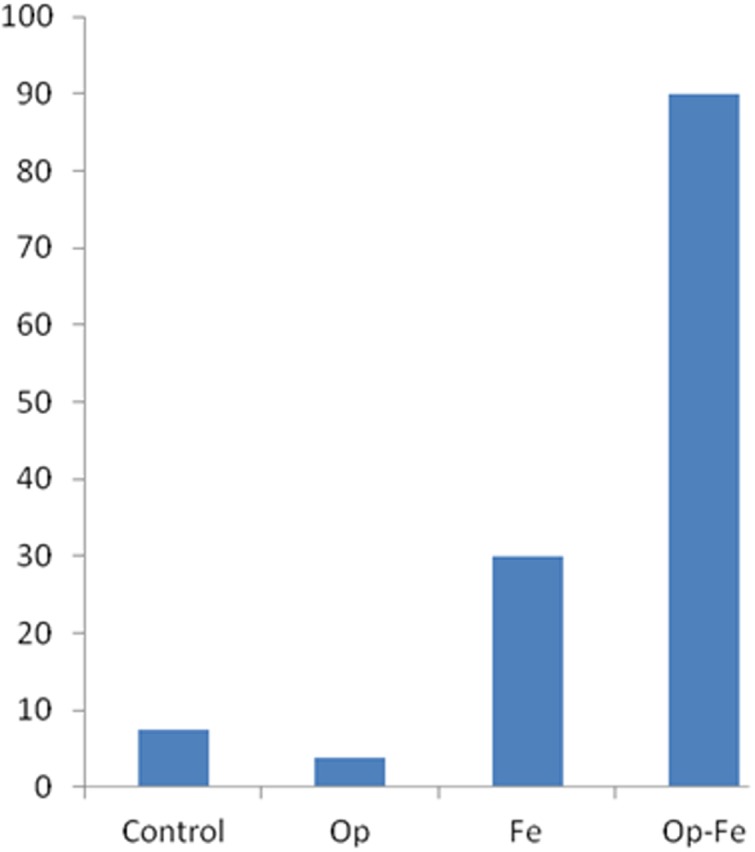
**Percentage of Rule II users in each condition**.

Finally, we classified a child as using Rule II if her or his predictions on at least 4 of 5 distance problems was correct, that is, child indexed that the side with its weight farther from the fulcrum would go down (Siegler and Chen, 1998). The chi-square test on the number of children who used Rule II indicated that it differed considerably between different conditions, χ^2^(3) = 45.38, *p* < 0.001. As shown in **Figure 4**, just few children in the *Control* and *Op* conditions could be classified as using Rule II, whereas about 30% of the children in the *Fe* condition used Rule II, which were significantly more than those who did so in the *Control* (*Z* = 1.96, *p* = 0.05) and *Op* conditions (*Z* = 2.42, *p* = 0.015). The Rule II users in the *Op-Fe* condition were significantly more than they were in the *Fe* condition (*Z* = 3.82, *p* < 0.001).

## Discussion

Consistent with previous findings that children under 8 years of age often solve the balance scale problems based on the Weight Rule (Siegler, 2005; Schapiro and McClelland, 2009), our study found that, when there is no feedback and opportunity to operate the scale (in the *Control* condition), the accuracy of responses in the weight and conflict-weight problems were obviously higher than they were in the other three types of problems, which means that children tend to use Rule I, the Weight Rule, to solve balance problems (Siegler, 2005). Children in the present study were shown a balance scale with a varying number of weights placed on pegs on each side, at varying distances from the fulcrum. So the Weight Rule means that children count the number of the weight objects to solve the weight problem (Halford et al., 2002; Schrauf et al., 2011). Younger children seemed have difficulty in understanding the role of distance when determining the movement of the balance scale; therefore, they were unable to solve the balance scale problems based on the distance from the fulcrum.

It is difficult for 5-year-olds to think about one question from multiple dimensions (Piaget et al., 2013). They are unable to solve the problems that contain conflict between different dimensions, but they can solve single-dimension problems and perform well in solving the weight problems. Though distance problems are single-dimension problems, as compared with weight problems, the former are more abstract (Bonawitz et al., 2012). Given the fact that in the balance scale task, while the weight of the objects will change with the number of objects, and the children’s perception toward fewer than four objects has already become mature (when they are very young) (Zhou, 2014), children will be relatively sensitive to the changes in weight, thus engaging in indexing by the number of objects. In addition, the Naïve theory has taught children above 4 years of age that the heavier a thing is, the more likely it will tip down (Schrauf et al., 2011). Therefore, they can successfully solve the weight problem. On the contrary, in solving the distance problem, as the distance from the fulcrum changes, children are required to answer which side will tip down. Obviously, to solve the distance problem, a child not only needs to consider the distance dimension, which varied horizontally, and predict the vertical movement of the two sides of the balance scale, but she or he also needs to understand the principle behind the lever; thus, it is difficult for younger children to solve the distance problem.

Some researchers have shown that, while providing feedback to children helps them solve the problem of the balance scale, the effect of feedback was mainly observed when children were repeatedly presented with the same type of problems, such as distance problems in a block (Siegler and Chen, 1998). We found that feedback can also help children acquire a higher level rule when different types of problems were arranged with the same frequencies in one block.

The performance curve in the present study showed that, in the no-feedback condition, children’s performance on the distance or conflict-distance problems did not increase with time. On the contrary, when feedback was provided, children’s performance increased with time. In previous studies, researchers presented children with one type of problem, and provided feedback that informed children of their correct or incorrect responses. When encountering the distance problem, for example, children do not know whether the far or near side from fulcrum will tip down. They may guess the near side, and the experimenter would show the correct answer, informing the children about whether they were correct or incorrect. If a child guessed wrong, she or he would give an opposite prediction in the next trial. This kind of feedback-based learning is easy for children, because they can easily learn from the feedback provided for the performance on the same type of problems, without the interference from the experience of other types of problems. In the present study, although there was interference from other types of problems, children still benefited from the feedback, reflecting that feedback has a fundamental effect on children’s rule learning. It should be emphasized that Siegler (1976) offered children feedback and gave them a chance to make an oral explanation, so it is unclear whether the increase in the use of Rule II was due to the effect of feedback or due to the oral explanation. Actually, the oral explanation can help children better understand the rules they have learned (Pine et al., 2004; Bonawitz et al., 2012). In the present study, we revealed that feedback without the oral explanation can also increase the use of Rule II.

Moreover, previous studies demonstrated that operation experience can also help children’s rule learning; but, how does it apply to children’s learning in the balance scale task? Further, is operation more or less effective than feedback? The present study found that feedback was more effective than operation was in rule learning. Specifically, without feedback, operation can improve accuracy by 15% in children solving the distance problem, but this increase did not achieve significance. According to the present data, we can tentatively conclude that operation experience cannot solely bring about significant improvement in children’s rule learning. This result was consistent with the result of Experiment 1 in Siegler’s (1976) study, in which it was demonstrated that if there is no explicit feedback for the questions or problems advanced by the experimenter, children’s rule learning cannot significantly be promoted, regardless of whether they are allowed to freely explore or passively observe the balance scales operated by the experimenter. Nevertheless, it is inappropriate to indicate that operation had no effect in children’s rule learning, because when feedback was accompanied with operation, children not only performed better in the one-dimension task (e.g., the distance problem), but their performance also improved in the double-dimension task, such as the conflict-distance problem.

Therefore, our study partially confirmed that operational experience can help children grasp high level rules better (Legare, 2014). With operational experience, children can more effectively and efficiently notice the distance changes, and better adjust their cognitive strategy when they receive negative feedback. Feedback played a key role in the learning process of children, given that children remembered each question and answer, which was enabled by inductive learning (Li et al., 2017). By manually operating the balance scale, children may efficiently strengthen their memory and facilitate their learning. In addition, children may be more likely to process the distance information because of their operational experience (Siegler and Chen, 1998). Specifically, children should know that the location of the weight used on both sides of the balance is different, and they should accurately place the weight on the correct locations. Thus, they should seriously process the distance information in order to receive a high score in solving the distance problem.

With reference to the cognitive limitation of young children (Piaget and Inhelder, 1969), children in the experimental groups still scored worse on all three types of conflict items, implying that they improved to the level of Rule II but not to that of Rule IV. Although children exhibited higher scores for the conflict-distance problems in the *Op-Fe* condition as compared to those in the other three conditions, the improvement might be simply due to the fact that children paid more attention to the distance dimension than they did in the *Control* condition. Only when they begin to compare the torques (number of weights multiplied by distance of those weights from the fulcrum) on each side of the scale, they can use Rule IV correctly to make their predictions on the balance task (Jansen and van der Maas, 2002; Siegler and Chen, 2002; Schapiro and McClelland, 2009).

## Conclusion

Both feedback and operational experience may help children to better understand the rule of balancing and use the high level rules; however, their functions are different. Operation experience helped children to precisely encode the distance information, while feedback allowed children to note the importance of the role of distance, and enabled them to use Rule II. The combination of feedback and operational experience will allow children to process the distance more accurately, and would thus lead them to more frequently attempt to use Rule II. In this way, they will attain a higher score in solving some double-dimension problems (such as the conflict-distance problems), reflecting the attempt to use Rule III. These findings extend our understanding of children’s rule learning, implying that feedback-based learning, combined with manual operation, is the most efficient method for facilitating rule learning in children, although feedback is more effective than operational experience is in this case.

## Author Contributions

Conceived and designed the experiments: FL and BC. Performed the experiments: LX and XY. Analyzed the data: LX. Wrote the paper: FL, LX, and BC.

## Conflict of Interest Statement

The authors declare that the research was conducted in the absence of any commercial or financial relationships that could be construed as a potential conflict of interest.
